# Implementing technologies to prevent medication errors at a high-complexity hospital: analysis of cost and results

**DOI:** 10.31744/einstein_journal/2019GS4621

**Published:** 2019-06-25

**Authors:** Renata Prado Bereta Vilela, Marli de Carvalho Jericó

**Affiliations:** 1 Faculdade Faceres, São José do Rio Preto, SP, Brazil; 2 Faculdade de Medicina de São José do Rio Preto, São José do Rio Preto, SP, Brazil

**Keywords:** Patient safety, Medication errors, Medication systems, hospital, Technology, Accident prevention, Costs and cost analysis, Segurança do paciente, Erros de medicação, Sistemas de medicação no hospital, Tecnologia, Prevenção de acidentes, Custos e análise de custo

## Abstract

**Objective:**

To calculate the cost and assess the results on implementing technological resources that can prevent medication errors.

**Methods:**

A retrospective, descriptive-exploratory, quantitative study (2007-2015), in the model of case study at a hospital in the Brazilian Southeastern Region. The direct cost of each technology was calculated in the drug chain. Technological efficacy was observed from the reported series of the indicator incidence of medication errors.

**Results:**

Thirteen technologies were identified to prevent medication errors. The average cost of these technologies per year in the prescription stage was R$ 3.251.757,00; in dispensing, R$ 2.979.397,10; and in administration, R$ 4.028.351,00. The indicator of medication error incidence decreased by 97.5%, gradually between 2007 to 2015, ranging from 2.4% to 0.06%.

**Conclusion:**

The average cost per year of the organization to implement preventive technologies in the drug chain totaled up R$ 10.259.505,10. There was an average investment/year of R$ 55,72 per patient and its association with smaller indicator of incidence of medication errors confirms a satisfactory result in this reported series regarding such investment.

## RESUMO

**Objetivo:**

Calcular o custo e avaliar os resultados da implantação de tecnologias que podem prevenir o erro de medicação.

**Métodos:**

Estudo descritivo-exploratório, retrospectivo (2007-2015), quantitativo, nos moldes de estudo de caso em instituição hospitalar da Região Sudeste do Brasil. Calculou-se o custo direto de cada tecnologia na cadeia medicamentosa. A eficácia das tecnologias foi verificada a partir da série histórica do indicador de incidência de erro de medicação.

**Resultados:**

Para prevenção do erro de medicação, foram identificadas 13 tecnologias. O custo médio/ano dessas tecnologias na etapa de prescrição foi R$ 3.251.757,00; na dispensação, R$ 2.979.397,10; e na administração, R$ 4.028.351,00. O indicador de incidência de erro de medicação apresentou queda de 97,5%, de forma gradual entre 2007 a 2015, variando de 2,4% a 0,06%.

**Conclusão:**

O custo médio/ano para a instituição das tecnologias preventivas na cadeia medicamentosa totalizou R$ 10.259.505,10. Houve investimento médio/ano de R$ 55,72 por paciente. Sua associação com a redução do indicador de incidência de erros de medicação na série histórica apresentada reitera um resultado satisfatório para tal investimento.

## INTRODUCTION

In taxonomy related to patient safety, adverse drug events (ADE) are defined as damage caused to patients by the medication.^(^ ^1^ ^)^ Adverse drug events are only considered medication errors when they can be avoided or prevented.^(^ ^2^ ^)^ In this way, medication error is understood as any event that can be avoided or prevented, and that can occur at any phase of drug therapy, whether or not it causes damage to the patient.^(^ ^3^ ^,^ ^4^ ^)^

Growing number of studies and epidemiological data demonstrate that medication errors are present in different situations of health care. A practice that is safe and free of damage is considered a global objective, as presented in the document, Global Patient Safety Challenge on Medication Safety, of the World Health Organization (WHO). Nevertheless, investments are necessary in the development of systems, practices, and technologies that can prevent errors and improve drug therapy.^(^ ^5^ ^)^

The definition of technologies in health is broad, and not restricted to pieces of equipment. It covers certain constituted elements of knowledge for generation and use of products, as well as for the organization of human relations through which the appropriate care and attention is given to the health of the population.^(^ ^6^ ^,^ ^7^ ^)^ Technologies may be classified as hard, represented by equipment; soft-hard, which includes structured knowledge (standards and protocols); and finally, soft, which is expressed by communication, by relations, and by associations.^(^ ^6^ ^)^

Technologies that can prevent medication error still face certain barriers to their implementation at healthcare organizations. One of them is its high cost.^(^ ^6^ ^,^ ^8^ ^)^ Nonetheless, it is necessary to consider its prevention benefits (intangible or immeasurable costs), and the cost of medication error itself.

Medication errors foster high costs to the healthcare system. Studies on this topic show low levels of evidence and a high variability of values.^(^ ^9^ ^)^ World Health Organization estimates that medication errors cost annually R$ 137 billion (US$ 42 billion).^(^ ^5^ ^)^ One of the first studies that focused on this theme, considered a methodological reference for current studies, showed ADE have an annual cost of R$ 10.992.800,00 (US$ 5.6 billion) and avoidable events (medication errors with damage) can reach R$ 5.496.400,00 (US$ 2,8 billion) for healthcare institutions.^(^ ^10^ ^)^

Medication errors, in addition to causing high costs, can also lead to disorders, such as changes in the therapeutic result of patients and increased morbidity and mortality,^(^ ^11^ ^,^ ^12^ ^)^ besides psychological problems for the professionals involved,^(^ ^13^ ^)^ among others. In this way, it is imperative that there be investments in prevention and that the cost be known, favoring the decision-making and the promotion of a patient safety culture.

## OBJECTIVE

To calculate the cost and evaluate the results of implementing technologies that can prevent medication error in a high complexity teaching hospital.

## METHODS

This is a retrospective, analytic, observational study (between 2007 and 2015). The context of the investigation was a special size (699 beds) philanthropic teaching hospital, with an average of 40,733 cases seen each year, 86,749 emergency care cases per year, and 19,193 hospital admissions of patients from the public and private health systems.

Data collection was conducted after its approval by the Research Ethics Committee (opinion 325.938), CAAE: 16212013300005415. The procedure for data collection had four stages.

In the first stage (from 2007 to 2015) - identification of technologies implemented at the organization, a meeting was held with nurses from the risk management area and the *Centro Integrado de Educação Permanente em Saúde* (CIEPS) [Integrated Center for Permanent Health Education], who had participated in the process of implementing prevention technologies in this period.

In the next stage, flowcharts of the subprocesses were drawn (prescription, dispensing and administration of drugs) of the medication flow, by means of Standard Operating Procedures (SOP) of the organization, in addition to indirect observation by the researcher. Technologies were included among the activities contained in the flowchart.

The third stage - validation of the flowcharts and technologies, was performed by 26 professionals who worked in the medication flow steps, including physicians, pharmacists, pharmacy technicians, nurses, nurse technicians and licensed practical nurses. These were randomly selected during the period of 2014 and 2015, from the areas of internal, surgical, emergency and intensive care medicine, and pediatrics. The validation process consisted of providing a few identification data, such as background, area of work, time worked in that area, and activity at the organization. The participant was also requested to estimate the time spent for a unit of activity done that would confirm if all the activities and technologies were present in the correct order in the flowcharts of the subprocesses of the medication flow. Finally, list the type of medication error that each technology could prevent. Validation had 73% (n=19) concordance, in which 60% (n=3) for medical prescription, 80% (n=8) for dispensing, and 73% (n=8) for administration of the medication. The differing ideas were analyzed, compared to the answers of other participants, and considered when in accordance with the practice. For the description of results, the preventive technologies were grouped on tables, according to the stages of the medication flow. The stages analyzed in this study were prescription, dispensing, and administration of drugs.^(^ ^14^ ^,^ ^15^ ^)^ Technologies that depend on equipment were classified as hard; soft-hard covered structured knowledge, standards, protocols, and knowledge; and soft technologies were characterized by interpersonal relations.^(^ ^6^ ^)^ After recognition and validation of the technologies, those that allowed measurement of their use, without apportionments, for the prevention of medication error were selected.

In the final stage - measurement of costs, the direct costing method was used to calculate all costs and expenses (fixed or variable), using as criterion values that could be directly appropriated to those that were being financed.^(^ ^16^ ^)^ Thus, the cost of human resources, based on multiplication of the time estimated (by the professionals that validated the flowcharts) using the mean number of times the activity is performed by the base salary of the professional. Material resources were calculated based on the cost of equipment and of materials necessary for their application, as well as the average quantity used per patient. The technologies that undergo depreciation had their calculation performed using the parameters of the depreciation table entitled, “Process of convergence of the municipal public accounts of the National Confederation of Municipalities, available at https://www.cnm.org.br/contadores/img/pdf/parte_2_depreciacao.pdf.

Data relative to costs were obtained from the following departments: Nursing management, hospital admission, hospitalization, accounting, financial superintendence, general storeroom, and information technology.

The unit costs were calculated for each technology per patient, as well as the costs within an annual projection. The present currency used was Reals (Brazil). Whenever there was a need to convert any currency, the Central Bank of Brazil was used, available at http://www4.bcb.gov.br/pec/conversao/conversao.asp. For currency conversion, the year of publication of the article that presented the value was used, except for the 1997 investigation, since the website only provides data as of 1999. For the calculation of the estimated cost of medication error, a study that estimated the cost of avoidable adverse events at a teaching hospital with 700 beds was used.^(^ ^10^ ^)^ This study was chosen due to the context similar to that of the current study, despite the fact that this study was carried out in 1997. The conversion was performed based on the year of the indicator and the cost of preventive technology, for a better comparison.

To evaluate the result of implementing preventive technology, the indicator medication error incidence was used, which is an equation proposed by the *Compromisso com a Qualidade Hospitalar* (CQH) [Commitment to Hospital Quality], an agency in which the organization participates. The indicator is represented by the ratio between the number of medication administration errors and number of patients/year, in which the value should be multiplied by one hundred.^(^ ^17^ ^)^ Collection of the indicator is performed manually and daily by the clinical nurses, by means of observation and notification of the nursing team in a separate spreadsheet and forwarded monthly to Nursing management.

For the tabulation and analysis of the study data, Excel software was used. The results were presented as frequency and proportions.

## RESULTS

To avoid medication error at the organization under study, professionals active in the medication flow identified 13 preventive technologies, namely training for nursing professionals and orientation program for the newly hired nursing team; use of the infusion pump; double checking of High Risk Medications (HRM); identification of routes of administration (labels with different colors); identification of the patient's bed (plate); identification of patient (bracelet); dispensing of medications (palmtop), and bar code reader; use of unit packages; organized medication kits in the operating room; identification of HRM (colored label); identification of HRM (colored plastic bag); and medical prescription (electronic).

These preventive technologies are distributed in each stage of the medication flow (Table 1) as follows: six (46.2%) prevent only the phase of medication administration; one (7.7%) prevents during the prescription and administration; five (38.4%) during dispensing and administration; and finally, one (7.7%) prevents all phases of the medication flow (prescription, dispensing and administration). When classifying type of technology, two (15.4%) are soft, one (7.7%) is soft-hard, and ten (76.9%) are hard.

**Table 1:** Cost per patient of medication error prevention technologies, according to stages of the medication flow

Medication Flow	Implementation	Classification	Technology	Cost of labor		Cost of material/equipment		Total cost	
				2014	2015	2014	2015	2014	2015
Administration	2011	Soft	Trainings	0.37	0.40	0.00	0.00	0.37	0.40
Administration	2011	Soft	Orientation	0.68	0.73	0.00	0.00	0.68	0.73
Administration	–	Hard	Infusion pump	1.08	1.31	12.23	15.23	13.31	16.54
Administration	2014	Soft-hard	Double checking	1.33	1.43	0.00	0.00	1.33	1.43
Administration	2012	Hard	Identification of routes	0.96	1.04	0.00	0.00	0.96	1.04
Administration	2013	Hard	Identification plates	2.52	3.98	0.20	0.28	2.72	4.26
Prescription and administration	2011	Hard	Identification bracelets	0.51	0.56	0.78	0.86	1.29	1.42
Dispensing and administration	2010	Hard	Electronic dispensing	1.95	2.13	3.63	3.52	5.58	5.65
Dispensing and administration	2013	Hard	Unit packages	0.49	0.50	1.83	2.09	2.32	2.59
Dispensing and administration	2013	Hard	Kits	7.45	7.43	0.53	0.46	7.98	7.89
Dispensing and administration	2012	Hard	Label for HRM	0.01	0.01	0.05	0.05	0.06	0.06
Dispensing and administration	2014	Hard	Bag for HRM	0.04	0.04	0.07	0.07	0.11	0.11
Prescription, dispensing and administration	2010	Hard	Electronic prescription	12.88	13.95	2.97	2.81	15.85	16.76
Total				30.27	33.51	22.29	25.37	52.56	58.88

HRM: high risk medication.

The cost with labor of the technologies per patient was R$ 30,27 in 2014, and R$ 33,55 in 2015, with an average cost of R$ 31,91, varying from R$ 0,01 to R$ 13,95. For the administration phase, R$ 7,92, on average, were invested per patient/year, for prescription and administration R$ 0,54; dispensing and administration R$ 10,03; and for prescription, dispensing, and administration R$ 13,41. As to the cost of labor related to classification of technologies, we obtained a mean cost of R$ 1,10 (3.4%) with the soft technologies; the soft-hard technologies cost, on average, R$ 1,38 (4.3%), and the hard technologies, R$ 29,42 (92.3%). Proportionally, the labor cost of the hard technologies was 88%, that is, more costly than soft-hard technologies, and 89% more costly than soft technologies.

The cost with material resources/equipment of the technologies per patient was R$ 22,29 in 2014, and R$ 25,37 in 2015, with an average cost of R$ 23,83, varying from R$ 0,00 to R$ 15,23. For the administration phase, on average, R$ 13,97 (58.6%) were invested per patient a year; for prescription and administration, R$ 0,82 (3.4%); dispensing and administration, R$ 6,16 (25.8%); and for prescription, dispensing, and administration R$ 2,89 (12.1%). As to the cost with material resources/equipment related to the classification of technologies, we found an average cost of R$ 0,00 (0%) with soft technologies, and R$ 23,84 (100%) with hard technologies. Proportionally, it can be stated that hard technologies were 100% more costly relative to material resources/equipment than the soft and soft-hard technologies were.

The total cost (cost of labor + cost of material/equipment) of technologies per patient was R$ 52,56 in 2014, and R$ 58,88 in 2015, with an average cost of R$ 55,72 per patient per year. The average cost of preventive technologies in administration was R$ 21,89 (39.3%) per patient; for prescription and administration, it was R$ 1,36 (2.4%); for dispensing and administration, R$ 16,18 (29.0%); and for prescription, dispensing, and administration, R$ 16,31 (29.3%). Regarding the type of technology, R$ 1,10 (1.9%) was invested in soft technologies; R$ 1,38 (2.5%) in soft-hard technology; and R$ 53,25 (95.6%) in hard technologies. Proportionally, the hard technologies were 91.2% more costly than soft and soft-hard technologies.

The average cost per patient in medication error prevention technologies was calculated, according to the stages of the medication flow (Table 2). For prescription (electronic), R$ 17,67 (31.7%) were invested; for dispensing (individualized system), R$ 16,19 (29.0%); and for administration R$ 21,89 (39.3%), totaling up R$ 55,75. During the study period, the mean number of patients seen per year was 184,027. Thus, the annual projection of the investment was R$ 10.259.505,10.

**Table 2:** Average cost per patient and annual projection of medication error prevention technologies, according to the stages of the medication flow

Stage of medication flow	Cost of labor		Cost of material		Total cost		Annual projection	
	R$	%	R$	%	R$	%	R$	%
Prescription	13,96	43.7	3,71	15.6	17,67	31.7	3.251.757,00	31.7
Dispensing	10,03	31.5	6,16	25.8	16,19	29.0	2.979.397,10	29.0
Administration	7,92	24.8	13,97	58.6	21,89	39.3	4.028.351,00	39.3
Total	31,91	100.0	23,84	100.0	55,75	100.0	10.259.505,10	100.0

As to the year of implementation of technologies, this occurred between 2010 and 2014, in which two (15.4%) were in 2010, three (23.1%) in 2011, two (15.4%) in 2012, three (23.1%) in 2013, two (15.4%) in 2014, and for one (7.6%) it was not possible to identify the year of implementation.

When listing the investment in technologies per year (Table 3), we noted a greater investment in 2010 (R$ 4.035.712,10; 39.5%), related to the implementation of the Hospital Information System (SIH - *Sistema de Informação Hospitalar*). The year with the lowest investment was 2012 (R$ 185,867.27; 2.7%), related to the implementing route identification and HRM identification per colored label. The projection of the total mean accrued investment was R$ 10.224.540,07/year.

**Table 3:** Indicator of incidence of medication errors, cost per patient and annual projection of costs of medication error prevention technologies

Year	Indicator %	Cost per patient (R$)	Annual projection (R$)	Cost of error/avoidable/year (R$)
2007	2.4	14,93	2.747.523,10	5.984.160,00^(^ ^10^ ^)^
2008	2.3	–	–	4.957.400,00^(^ ^10^ ^)^
2009	2.1	–	–	6.541.360,00^(^ ^10^ ^)^
2010	1.5	21,93	4.035.712,10	4.873.120,00^(^ ^10^ ^)^
2011	0.9	2,31	425.102,37	4.663.120,00^(^ ^10^ ^)^
2012	0.5	1,01	185.867,27	5.250.280,00^(^ ^10^ ^)^
2013	0.29	13,89	2.556.135,00	5.720.120,00^(^ ^10^ ^)^
2014	0.08	1,49	274.200,23	6.557.600,00^(^ ^10^ ^)^
2015	0.06	–	–	7.435.680,00^(^ ^10^ ^)^
Total	1.1	55,56	10.224.540,07	47.319.720,00

Source: Bates DW, Spell N, Cullen DJ, Burdick E, Laird N, Petersen LA, et al. The costs of adverse drugs events in hospitalized patients. Adverse Drug Events Prevention Study Group. JAMA. 1997;277(4):307-11.^(^^10^^)^

During the period from 2007 to 2015, the indicator incidence of medication error was 1.1%, whereas in 2007 it was 2.4%, and in 2015, 0.06%. From 2007 to 2010, year of the implementation of the first preventive technologies, there was a drop of the indicator by 37.5%, from 2010 to 2015; moreover, 100% of technologies had already been implemented when the 96% plunge in the indicator occurred. During the period of 2007 to 2015, the indicator declined by 97.5%.

## DISCUSSION

Medication error is a global issue, since the possibility of harm to the patient generates concerns for health-related agencies. In 2017, the third global patient safety challenge was launched by the WHO, geared towards medication without harm. This challenge foresees a reduction by 50% in the rate of medication-related severe and avoidable harm in the next 5 years.^(^ ^5^ ^)^ This challenge focuses on the improvement of medication systems and practices; hence, it becomes of utmost importance to be familiar with the technologies that helped preventing medication errors and their costs.

Currently, technology is an indispensable input, both in quantity and in quality in the process of health-related work, with repercussions in the quality of care offered to patients.

In the findings of this study, the greater use of hard technology (76.9%) in the medication flow is noteworthy. A study on the importance of the use of technology for patients’ safety^(^ ^18^ ^)^ analyzed four of them for reduction and prevention of medication errors at hospitals. Among these technologies, three were classified as hard, and were implemented by the organization analyzed in the current study, namely electronic medical prescription, bar codes, and infusion pumps. The other technology, with soft-hard characteristics, was not implemented (unit dose medication dispensation system), since this system is considered most appropriate in the prevention and reduction of medication error. At the organization of this study, the present dispensing system is individualized, that is, an intermediate system between the collective and per unit dosing. One can see that incorporation of hard technology demands great investments on the part of the organization, not reflecting the reality of most Brazilian hospitals, in which resource limitation is apparent, and costs play an important role in the allocation of these resources.

Currently, there is a certain glamour regarding electronic prescription, a hard technology incorporated since 2010 by the hospital of this study. This innovation corroborates an investigation carried out at a large-scale hospital, also equipped with computerized prescription systems, which demonstrated that this was a great advancement for the strategies of minimizing medication errors.^(^ ^19^ ^)^ Nevertheless, it is necessary that there be a certain involvement of the professionals, so that the use of technology as a barrier be effective.

As to soft-hard technology, which involves a care method,^(^ ^6^ ^)^ there was identification of one of them − double checking, which happens at the time of administration of medication, considering the degree of risk and/or of the complexity of the action. Exploratory bibliographic research points to double checking as the method adopted by some organizations during dispensing and administration of medications, principally the HRM, in which two professionals check the data before the medication is administered to the patient, reducing the chances of error.^(^ ^20^ ^)^

In this study, two soft technologies were identified, that is, the live work,^(^ ^6^ ^)^ and are related to continued education and the process of integration of the nursing team. The educational aspects in health allow the construction of new knowledge, leading to a practice consistent of preventive and humanized behavior.^(^ ^21^ ^)^ A study points to the improvement of care after an education measure with the professionals.^(^ ^22^ ^)^ On the other hand, difficulties in human relationships have been pointed out,^(^ ^23^ ^)^ such as disadvantages in the use of hard technologies, making them cold, objective, and individualistic, and distancing the interactions between the professional and the patient, inherent to the act of providing care. Thus, there is a certain status in hard technology related to technological innovation in detriment of the soft technology, which exerts a strong influence on the practice of the health professional, where the perception of the needs for care of the patient, integrated with the use of different types of technologies, comprise a guarantee of patient safety.

Comparing the projection of the cost in technologies for the prevention of medication error with the projection of the cost of the medication error, we note that the value of wastefulness (cost of the preventable adverse event) is higher than R$ 47.319.720,00 of the investment (cost of the preventive technologies) R$ 10.224.540,07. Therefore, prevention proves to be less costly than the medication error, besides the benefits of prevention that go beyond the costs, given that the medication error also includes the increase in morbidity and mortality of the population.^(^ ^24^ ^)^

The technologies proved to be effective, since there was a drop by 97.5% in the indicator incidence of medication error. However, a study conducted at a teaching hospital with the objective of characterizing medication errors, and identifying causes of and actions after their occurrence, points to subnotification of this indicator as a problem. This is a reality, since we are still inserted in a punitive culture that relates error to human failures, and not to the processes.^(^ ^25^ ^)^ Hence, there is a need for a greater incentive to notify.

This study presents with limitations as to costs, since only direct costs and those of technologies capable of performing the calculation without apportionments, enabling, in this form, other costs not being considered in the total calculation. For the cost of labor, it was requested of the professionals involved in the action that they estimate the time of activity, instead of the researcher timing it. Another limitation is the use only of the indicator to verify the efficacy of the technology, since there was underreporting, possible not reflecting the real situation.

There are advancements in this study relative to the visualization of an economic panorama regarding the issue of prevention of medication error, since the high cost of prevention is frequently reported, but it is not measured. A second advancement is the comparison between the cost of the preventable adverse events and the implementation of the preventive technologies for this event, demonstrating the difference in values between waste and investment.

## CONCLUSION

This study highlights the importance of knowing the costs for investing in technological resources that can prevent medication errors. The investment in technologies that can prevent patient medication was estimated, and the annual projection of this investment was made for the organization. The technologies proved effective since there was a significant drop in the quality indicator of medication error incidence.
